# Association of Methyl Donor Nutrients’ Dietary Intake and Cognitive Impairment in the Elderly Based on the Intestinal Microbiome

**DOI:** 10.3390/nu16132061

**Published:** 2024-06-28

**Authors:** Qianqian Chen, Rui Fan, Lixia Song, Shuyue Wang, Mei You, Meng Cai, Yuxiao Wu, Yong Li, Meihong Xu

**Affiliations:** 1 Department of Nutrition and Food Hygiene, School of Public Health, Peking University, Beijing 100191, China; 2211210120@stu.pku.edu.cn (Q.C.); fanruirf@bjmu.edu.cn (R.F.);; 2 Beijing Key Laboratory of Toxicological Research and Risk Assessment for Food Safety, Peking University, Beijing 100191, China

**Keywords:** cognitive impairment, gut microbiome, one-carbon metabolism, nutrition, aging

## Abstract

Globally, cognitive impairment (CI) is the leading cause of disability and dependency among the elderly, presenting a significant public health concern. However, there is currently a deficiency in pharmacological interventions that can effectively cure or significantly reverse the progression of cognitive impairment. Methyl donor nutrients (MDNs), including folic acid, choline, and vitamin B12, have been identified as potential enhancers of cognitive function. Nevertheless, there remains a dearth of comprehensive research investigating the connection between the dietary intake of MDNs and CI. In our study, we comprehensively assessed the relationship between MDNs’ dietary intake and CI in older adults, utilizing 16S rRNA gene sequencing to investigate the potential underlying mechanisms. The results showed an obvious difference in the methyl-donor nutritional quality index (MNQI) between the dementia (D) group and the dementia-free (DF) group. Specifically, there was a lower MNQI in the D group than that in the DF group. For the gut microbiome, the beta diversity of gut flora exhibited higher levels in the high methyl-donor nutritional quality (HQ) group as opposed to the low methyl-donor nutritional quality (LQ) group, and lower levels in the D group in comparison to the DF group. Subsequently, we performed a correlation analysis to examine the relationship between the relative abundance of microbiota, the intake of MDNs, and Montreal Cognitive Assessment (MoCA) scores, ultimately identifying ten genera with potential regulatory functions. Additionally, KEGG pathway analyses suggested that the one-carbon metabolism, chronic inflammation, and DNA synthesis potentially serve as pathways through which MDNs may be promising for influencing cognitive function. These results implied that MDNs might have the potential to enhance cognitive function through the regulation of microbiota homeostasis. This study offers dietary recommendations for the prevention and management of CI in the elderly.

## 1. Introduction

It is expected that dementia prevalence will rise as the global population ages, with projections indicating a tripling in the number of individuals living with this condition by the year 2050 from the estimated 47 million in 2015 [1]. Cognitive impairment (CI) includes both mild cognitive impairment (MCI) and dementia, the latter characterized by a more pronounced deterioration in cognitive abilities that hinders daily functioning and social engagement [2]. Long-term caring for individuals with CI is a significant burden on patients, their families, and society. The estimated global cost of dementia in 2015 was USD 818 billion, a figure that is expected to rise as the prevalence of dementia increases [3]. This underscores the growing urgency of dementia as a significant global public health concern. Historically, dementia has been regarded as an incurable disease. However, emerging evidence suggests that a significant proportion of dementia cases may be preventable [4]. MCI represents a transitional stage between normal cognitive function and dementia, often serving as an early indicator of the latter [5]. Consequently, early intervention has emerged as a promising approach for the prevention and management of dementia. Several studies have provided evidence suggesting that dietary patterns may confer protective benefits against cognitive decline, particularly in individuals with MCI who undergo intervention [6]. Furthermore, lower serum folate levels [7,8] and higher homocysteine levels [9,10] have been linked to an increased risk of conversion from any type of MCI to all-cause dementia, a relationship that is associated with one-carbon metabolism.

One-carbon metabolism (OCM) has been identified as playing a significant role in the initiation and progression of CI [11,12]. OCM is a complex system of biochemical reactions that supply methyl donors with diverse biosynthetic pathways, supporting various physiological functions in the brain such as DNA synthesis, neurotransmitter synthesis, epigenetic regulation, and antioxidant defense mechanisms. Additionally, OCM serves as a pivotal point connecting multiple pathways [13]. The consumption of methyl donor nutrients (MDNs) such as protein, folate, choline, betaine, vitamin B2 (VB_2_), VB_6_, VB_12_, and zinc plays a crucial role in OCM [14,15]. Inadequacies in any of these nutrients can interfere with the intricate regulatory system responsible for maintaining the OCM, resulting in impairments in brain function [16]. Recent studies have consistently reported the potential therapeutic effects of MDNs in CI. B vitamins, particularly folate, VB_6_, and VB_12_, play a role in homocysteine metabolism and can decrease the risk of CI [17,18]. Additionally, choline supplementation has been shown to elevate brain acetylcholine levels and suppress neuroinflammation, leading to enhancements in learning and memory [19]. Betaine has been found to inhibit the overactivation of hippocampal microglia and decrease oxidative stress, thereby serving as a preventive measure against CI [20]. A deep understanding of the correlation between MDNs and CI may provide valuable insights into effective dietary strategies for preventing and managing dementia. Nevertheless, there is a lack of research that has thoroughly investigated the relationship between MDNs and CI.

The gut microbiota, a crucial element of the gut–brain axis, play a significant role in modulating cognitive functions through various mechanisms such as modulating neurotransmitter synthesis and metabolism, as well as regulating inflammatory and immune responses [21]. Recent research has increasingly demonstrated a correlation between gut microbial imbalance and the onset of CI [22]. While a definitive standard for gut microbiota homeostasis remains elusive, individuals with chronic illnesses often exhibit an elevated presence of pro-inflammatory bacteria and a diminished presence of beneficial bacteria. Patients with Alzheimer’s disease (AD), for example, commonly display dysbiosis in their gut microbiota, characterized by the reduced abundance and diversity of flora, heightened levels of pro-inflammatory bacteria, and an elevated Firmicutes/Bacteroidetes ratio [21,23,24]. Prior research has indicated that diets deficient in folate and VB_12_ may lead to a reduction in B vitamin-producing bacteria and an increase in inflammation-associated bacteria [25]. Thus, we posited that integrated MDNs may exert an influence on the gut microbiota, thereby potentially impacting CI.

To illustrate the relationship between dietary MDNs and CI in the elderly, and if dietary MDNs may affect CI, we systematically assessed the association between the dietary intake of MDNs and CI in the elderly, and explored the potential mechanisms based on the intestinal microbiota. This study has the potential to propose intervention strategies aimed at preventing CI in the elderly through a nutritional lens, as well as offering novel insights for the targeted manipulation of the intestinal microbiota.

## 2. Methods

### 2.1. Study Population

A cohort of 301 older adults aged 60–70 years was enrolled in the TALENTs trial (Targeting Aging and Longevity with Exogenous Nucleotides), a pragmatic four-month prospective single-center randomized controlled trial. Research protocols have been reported [26]. Participants in the study were administered the Montreal Cognitive Assessment (MoCA) to assess cognitive health, with a MoCA score of 18 or lower indicating membership in the dementia (D) group, while those scoring above 18 were categorized as belonging to the dementia-free (DF) group [27]. Demographic characteristics and medical history pertaining to chronic diseases were obtained through the administration of a questionnaire. Subsequently, fecal samples from all participants were collected, processed, and preserved at a temperature of −80 °C for future analysis. A total of 290 valid fecal samples were acquired, corresponding to the inclusion of 290 individuals in the ultimate analysis. All participants provided written consent by signing an informed consent form. The TALENTs trial has been approved by Peking University’s Biomedical Ethics Committee (IRB00001052-21114) and registered by Clincal Trials.gov (NCT05243018).

### 2.2. Assessment of Methyl Donor Nutrients Dietary Intake

The Photo-Assisted Dietary Intake Assessment (PAD) method was utilized to gather dietary intake data for MDNs. Previous research has demonstrated the accuracy and feasibility of the PAD method, particularly in its application to elderly populations [28]. Participants underwent standardized training and received real-time professional guidance. Subsequently, photographs of participants’ meals were taken before and after consumption over a period of three consecutive days to assess intake of both regular and additional foods. Subsequently, we determined the mean daily consumption levels of protein, folate, choline, riboflavin, VB_6_, VB_12_, betaine, and zinc. Subsequently, we calculated the methyl-donor nutritional quality index (MNQI) by considering the intake of these eight MDNs in accordance with the Chinese Dietary Reference Intakes (version 2023), in order to comprehensively evaluate the dietary intake of MDNs [29]. The methodology utilized in this study was derived from a prior research investigation that demonstrated a notable association between the MNQI and OCM, thus validating the MNQI as a reliable instrument for assessing the nutritional quality of dietary MDNs sources in a comprehensive manner. As per the findings of the study, an MNQI score equal to or exceeding 6 is classified as high methyl-donor nutritional quality (HQ), while a score below 6 indicates low methyl-donor nutritional quality (LQ) [29].

### 2.3. Analysis of Intestinal Flora Test

#### 2.3.1. 16S rRNA Gene Sequencing Analysis

We extracted fecal microbial DNA using the MGIEasy fecal genomic DNA (meta) extraction kit (BGI, Shenzhen, China). Subsequently, we used the primers PF5′-ACTCCTACGGGGAGGCAGCAG-3′ and PR5′-GGACTACNNGGGGTATCTAAT-3′ on the polymerase chain reaction (PCR) amplification of the bacterial V3–V4 highly variable region of the 16SrRNA gene. The PCR amplification products were purified and dissolved in Elution Buffer using Agencourt AMPure XP magnetic beads (Beckman Coulter, UK) and labeled to complete the library construction. The fragment range and concentration of the libraries were detected using an Agilent 2100 Bioanalyzer (Agilent, CA, USA). The qualified libraries were sequenced on the MGISEQ-2000 platform (BGI, Shenzhen, China) according to the size of the inserted fragments and the paired ends were sequenced. The raw data underwent filtering and splicing through the utilization of FLASH (Fast Length Adjustment of Short reads, v1.2.11), which facilitated the assembly of paired reads derived from double-end sequencing into a unified sequence based on overlap relationships to acquire Tags within the high-variance region. Subsequently, USEARCH (v7.0.1090) was employed to amalgamate the spliced Tags, which were then clustered at a 97% sequence similarity threshold to produce Operational Taxonomic Units (OTUs), with chimeric sequences being eliminated through the application of UCHIME (v4.2.40). After obtaining the OTU representative sequences, the OTU representative sequences were compared with the Ribosomal Database Project (RDP, http://rdp.cme.msu.edu/, accessed on 20 April 2024) for species annotation by RDP classifier (v2.2) software, with the confidence threshold set to 0.6.

#### 2.3.2. Bioinformatics Analysis

In order to study species diversity within one sample, Alpha Diversity analysis was performed, and The Alpha Diversity Index at the OTU level was calculated using mothur software (v1.31.2), including the Chao1 index, ACE index, and Shannon index. An analysis of the beta diversity was performed to compare the diversity of species between the samples, and the 16S rRNA gene beta diversity was analyzed using QIIME (v1.80) software. A predictive functional analysis of microbiota was performed using PICRUSt2 (v2.3.0-b) software to relate species to their functions.

Intergroup Venn diagrams were plotted to illustrate the overlap of OTUs between groups using the VennDiagram package in R (v3.4.1). The beta diversity was expressed as unweighted and weighted UniFrac distances and tested for intergroup differences using ANOSIM with the QIIME (v1.80) software. In addition, Partial Least Squares Discriminant Analysis (PLSDA) was performed using the mixOmics package to assess differences in the beta diversity. The pheatmap package facilitated the correlation of heatmap clusters while the Wilcoxon rank sum test was also utilized to determine differences in microbial abundance among taxa. Differences in microbial abundance were used to identify important species or functions.

#### 2.3.3. Statistical Analysis

The values of the numerical variables are given as the mean + standard deviation, and categorical variables are given as percentages. Nonparametric tests (including Wilcoxon rank-sum tests and Mann–Whitney U tests), independent sample *t*-tests, and Pearson’s chi-square tests were conducted to assess the differences between groups. Correlations between the numerical variables were analyzed using Spearman’s correlation analysis. We performed all statistical analyses using R (v3.4.1). Statistical significance was defined as *p* less than 0.05.

## 3. Results

### 3.1. Demographic Data for D Group and DF Group

The distribution of the general demographic characteristics for the D and DF groups was examined and no significant differences were found in terms of gender, age, education level, living alone or not, or body mass index (BMI), and the history of chronic diseases between the two groups is shown in Table 1.

The differences in gender, highest educational level, living alone or not, BMI, and chronic disease history between groups were analyzed by Pearson’s chi-square test. The differences in age were examined by an independent samples *t*-test.

### 3.2. Correlation between Dietary Intake of MDNs and Cognitive Function

The distribution of the MNQI was examined in both the D and DF groups. Table 2 illustrated the disparities in the median dietary intake of MNQI between the D and DF groups. Compared to the DF group, the D group scored significantly lower on the MNQI (*p* < 0.05), with notable differences observed in the protein, choline, riboflavin, and zinc intake compared to the DF group (*p* < 0.05). Furthermore, there was a noticeable decrease in the intake of folate, VB_6_, VB_12_, and betaine among participants in the D group. This suggests a potential correlation between the intake of methyl-donor nutrients (MDNs) and cognitive function, with a higher MDN intake potentially benefitting cognitive function.

### 3.3. Potential Role of Intestinal Flora in the Association between MDNs and Cognitive Function

#### 3.3.1. Differences in Intestinal Flora between Groups D and DF

**Figure 1:**
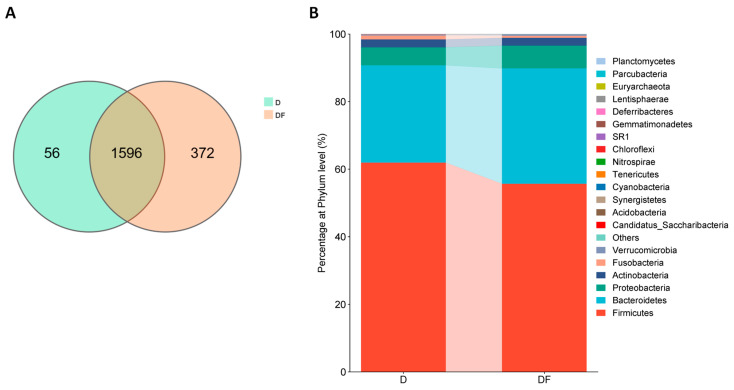
Distribution of identified intestinal flora in dementia (D) and dementia-free (DF) groups. (**A**) Venn diagram showing the distribution of OTUs between D and DF group. (**B**) Intestinal flora composition of the two groups at the phylum level.

To determine whether dietary MDNs affect cognitive function by the intestinal flora, a comparison of the microbial composition of groups D and DF was conducted. The findings revealed a total of 1652 operational taxonomic units (OTUs) in the D group and 1968 OTUs in the DF group, with 1596 OTUs shared between both groups (Figure 1A). For the phylum level, the composition of the intestinal flora in both groups included 28 phyla, with Firmicutes, Bacteroidetes, Proteobacteria, Actinobacteria, Fusobacteria, and Verrucomicrobia being the dominant phyla. However, there were variations in the distribution of these phyla between the two groups. In comparison with the DF group, the D group showed a higher ratio of Firmicutes to Bacteroidetes (Figure 1B).

**Figure 2:**
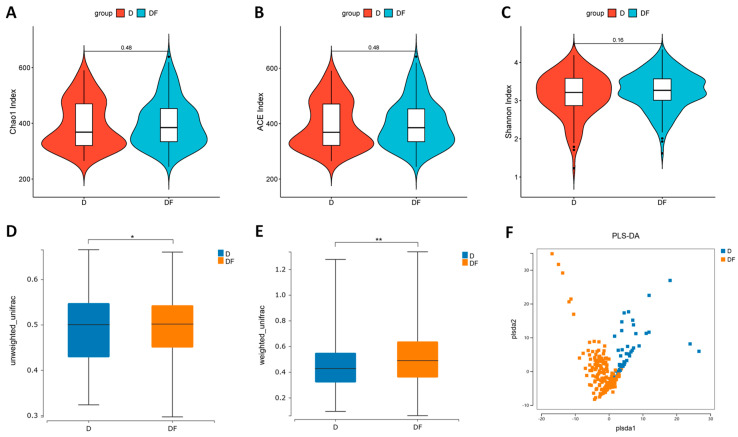
Comparisons of intestinal flora diversity between dementia (D) and dementia−free (DF) groups. (**A**) Chao1 index, (**B**) ACE index, and (**C**) Shannon index for assessing alpha diversity. *p*−value indicated differential clustering assessed by Wilcoxon rank−sum test. The beta diversity based on (**D**) unweighted and (**E**) weighted UniFrac distances analysis between the two groups. (**F**) PLS−DA analysis of intestinal flora between the two groups. *p*-value indicated differential clustering assessed by ADONIS test. * *p* < 0.05, ** *p* < 0.001.

Furthermore, an evaluation was conducted to compare the gut flora diversity between the two groups. The alpha diversity of the flora was assessed utilizing Chao1, ACE, and Shannon indices. D and DF groups showed no statistically significant variances in alpha diversity (*p* > 0.05, Figure 2A–C). The beta diversity index was utilized to assess the variation in species abundance distribution between the groups. The findings indicated a notable divergence in the composition of intestinal flora between the D and DF groups (Figure 2E,F), with the D group exhibiting lower beta diversity compared to the DF group (*p* < 0.05, Figure 2D,E). Therefore, there is a notable discrepancy in the abundance distribution of intestinal flora between the two groups. Specifically, patients with dementia exhibit decreased diversity in their intestinal flora, meaning specific alterations in the distribution of abundance of certain flora.

#### 3.3.2. Relationship between MDNs’ Intake and Intestinal Flora

**Figure 3:**
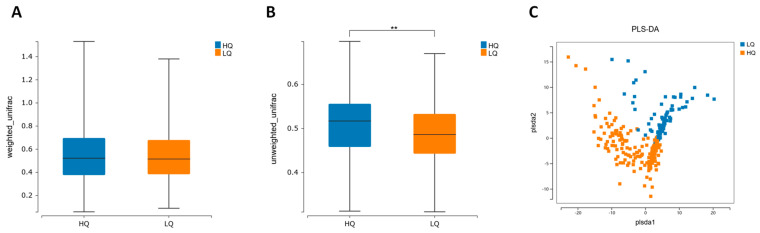
Comparisons of intestinal flora diversity between the LQ and HQ groups. The beta diversity based on (**A**) unweighted and (**B**) weighted UniFrac distances analysis between HQ and LQ group. (**C**) PLS−DA analysis of intestinal flora between HQ and LQ group. ** *p* < 0.001.

In this study, we employed beta diversity indices to assess the impact of dietary MDNs on the intestinal flora composition. The results demonstrated a significant difference in the distribution of intestinal flora abundance between the LQ and HQ groups, with the LQ group exhibiting lower beta diversity compared to the HQ group (*p* < 0.001, Figure 3A). Furthermore, the findings indicate that altering the abundance distribution of gut flora may have a close relationship to cognitive function, particularly given the lower beta diversity observed in the D group.

**Figure 4:**
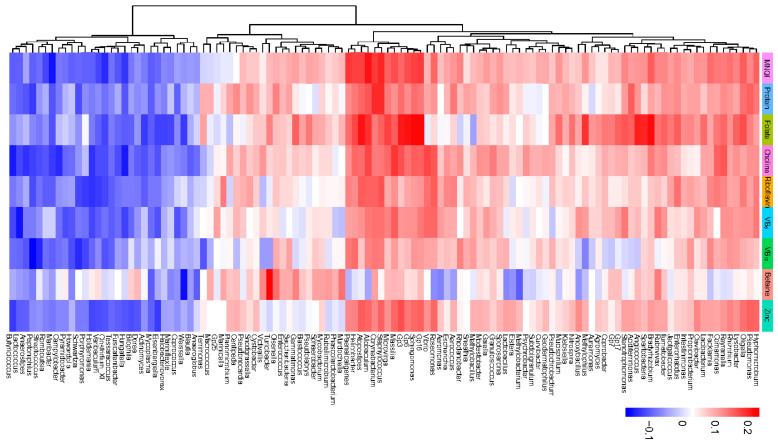
Heatmap clustering plot of correlations between the relative abundance of the initially screened genera and MDNs’ intake. Red: correlation is positive, blue: correlation is negative.

Subsequently, in order to investigate the relationship between the specific gut microbiota and MDNs, the relative abundance of gut microbiota was analyzed at the genus level. We then conducted a preliminary screening to identify gut microbiota influenced by micronutrient-dense nutrients, based on the presence of statistically significant differences in the correlation between the genera and MNQI or MDNs’ intake. Given the necessity of incorporating a greater number of potential flora in the screening phase to facilitate a broader selection for subsequent comprehensive investigations, it was determined that a statistical divergence in flora screening occurred at a significance level of *p* < 0.1. Figure 4 illustrates the initial screening of 115 candidate genera, which were subsequently categorized into two clusters based on their correlation with MDNs’ intake. Specifically, 85 genera exhibited a positive correlation with MDNs’ intake, while 30 genera showed a negative correlation. Among these genera, Gp16, Sphingomonas, Gp6, and Allobaculum exhibited significant positive correlations with the intake of MDNs, particularly demonstrating strong associations with the MNQI and folate intake. Conversely, genera such as Clostridium_XI and Maribacter displayed notable negative correlations with the MNQI. Furthermore, a consistent trend was observed where the MNQI and MDNs’ intake influenced the relative abundance of these genera, either up-regulating or down-regulating their presence.

#### 3.3.3. Screening for Specific Genera That May Mediate the Association of MDNs with Cognitive Function

In order to investigate potential genera that may play a role in the relationship between MDNs and dementia, we conducted an analysis of the correlation between MoCA scores and the relative abundance of the 115 genera initially identified. It was determined that a statistical divergence at a significance level of *p* less than 0.1 would determine statistical differences during the screening phase for the aforementioned reasons. Table 3 displayed the screening results of 10 potential genera, of which 7 were identified as having cognitive benefits. These genera, including Victivallis, Turicibacter, Phascolarctobacterium, Snodgrassella, Terrimonas, Planomicrobium, and Centipeda, exhibited a positive correlation in relative abundance with both MoCA scores and the intake of MDNs. Additionally, MoCA scores and the intake of MDNs were negatively correlated with the relative abundance of three cognitively harmful genera (Porphyromonas, Peptoniphilus, and Howardella). These findings imply that MDNs may have a significant impact on cognition through these genera. Thus, the study implied that dietary MDNs may improve cognitive function to some extent.

#### 3.3.4. Predicting Possible Mechanisms by which the Screened Genera Play a Role in Mediating the Association of MDNs with Cognitive Function

**Figure 5:**
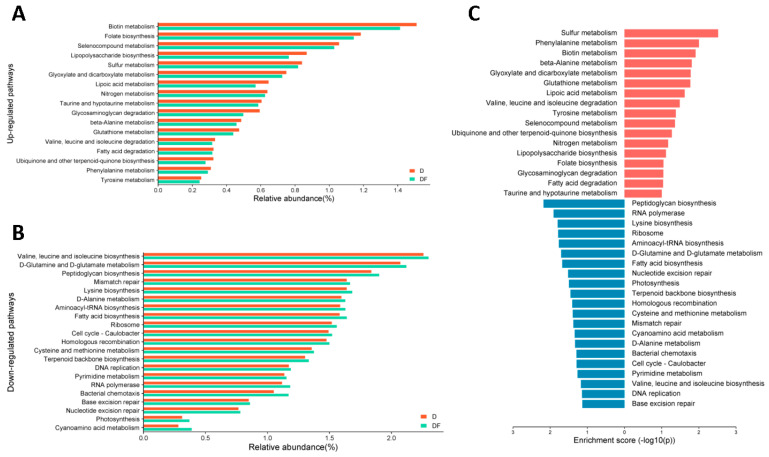
Significant differences between the D and DF groups at the third level of the KEGG pathway, *p* < 0.1. Compared with the DF group, the relative abundance of (**A**) up−regulated pathways and (**B**) down−regulated pathways of the KEGG pathway’s functional genes in D group. (**C**) Differences in the enrichment level of the KEGG pathway’s functional genes. Up−regulated functional genes in the D group compared to the DF group are labeled by red and down−regulated by blue.

Finally, we performed PICRUSt functional prediction analysis on the obtained 16S rRNA sequences to determine the enrichment of functional genes in the KEGG pathway, in order to speculate the possible mechanisms by which the screened intestinal flora play a role in mediating the effects of MDNs on dementia. Due to the limited sample size, *p* < 0.1 was considered statistically different. The findings indicated a significantly higher relative abundance of genes associated with 17 pathways, such as Sulfur metabolism, Phenylalanine metabolism, Biotin metabolism, beta-Alanine metabolism, Glyoxylate and dicarboxylate metabolism, and Glutathione metabolism, in the D group compared to the DF group. The pathways that were predominantly enriched among the up-regulated genes included Biotin metabolism, Folate biosynthesis, Selenocompound metabolism, Lipopolysaccharide biosynthesis, and Sulfur metabolism. In comparison to the DF group, the D group exhibited a significantly lower relative abundance of genes involved in 21 pathways, such as Peptidoglycan biosynthesis, RNA polymerase, Lysine biosynthesis, Ribosome, Aminoacyl-tRNA biosynthesis, D-Glutamine and D-glutamate metabolism, Fatty acid biosynthesis, and Nucleotide excision repair. Among the down-regulated pathways, Valine, leucine and isoleucine biosynthesis, D-Glutamine and D-glutamate metabolism, Peptidoglycan biosynthesis, Mismatch repair, and Lysine biosynthesis were mainly enriched (Figure 5).

## 4. Discussion

OCM plays a crucial role in preserving brain health. The inadequate consumption of dietary folate and VB_12_ has been linked to disturbances in OCM, leading to the dysregulation of the methylation pathway, increased oxidative stress, and enhanced protein deposition, ultimately hastening the development and advancement of CI [11]. Through interactions within the OCM pathway, methyl donors regulate metabolism, immune responses, and epigenetic events in animals [30]. Consequently, it is imperative to thoroughly investigate the association between the overall intake of MDNs and health outcomes. This study represents the first comprehensive assessment of the relationship between the MDNs’ intake and cognitive function. The findings revealed a significant correlation between cognitive function and dietary MDNs in older adults, suggesting that the intake of these nutrients may have a positive impact on the cognitive health status.

There is growing evidence linking imbalances in gut microbiota to the onset of neurodegenerative diseases [22], including AD [31], Parkinson’s disease [32], and multiple sclerosis [33]. Individuals with dementia have been observed to display dysbiosis in their gut microbiota, characterized by diminished abundance and diversity of flora [34]. Compared with those without dementia, individuals with dementia had significantly less gut flora beta diversity, with distinct differences in the flora composition and abundance between the two groups, consistent with previous research [35]. Additionally, dietary factors can influence the composition and function of gut microbiota, impacting the metabolic, immune, and neurological functions of the host through metabolically active substances that may contribute to the etiology and pathogenesis of CI [36,37,38]. Moreover, it suggested that diets enriched with MDNs may alter the intestinal environment and change the composition and distribution of the intestinal flora. Our research revealed a notable disparity in the beta diversity of intestinal flora between individuals with a low versus high MDN intake. Consequently, our findings propose that individuals with CI may exhibit distinct changes in their gut microbiota, and that dietary MDNs may influence cognitive function through the modulation of host gut–brain axis signaling [39].

Subsequently, utilizing correlation analysis, we identified particular genera that exhibited a significant association with both the intake of MDNs and the MoCA scores. Our findings suggest that heightened levels of Victivallis, Turicibacter, Phascolarctobacterium, Snodgrassella, Terrimonas, Planomicrobium, and Centipeda may potentially enhance cognitive function, and that the consumption of substantial quantities of MDNs could potentially elevate their prevalence. On the other hand, the elevated presence of Porphyromonas, Peptoniphilus, and Howardella has been linked to impaired cognitive function, whereas the consumption of substantial quantities of MDNs may reduce their prevalence, consequently enhancing cognitive well-being. Research on the impact of microbes on host health is providing insight into the roles of specific bacterial genera. For instance, a study examining Victivallis, a bacterium believed to have cognitive benefits, discovered that older adults with preclinical AD had lower levels of Victivallis in their gut microbiota compared to those without the disease, particularly in individuals testing positive for amyloid-β (Aβ), a pathological marker of AD [40]. A subsequent cross-sectional study involving individuals aged 70 and older revealed a decrease in Victivallis relative abundance among those with MCI [41]. The findings of this study align with previous research indicating a potential association between Turicibacter, known for its role in heightened bile acid production, and the disruption of bile acid homeostasis in the pathogenesis of AD, suggesting a possible neuroprotective effect of Turicibacter [42,43]. Studies have indicated that the screen-detected pathogenic bacterium Porphyromonas has the ability to colonize the brain and induce neuroinflammation, resulting in elevated levels of Aβ1-42, a protein associated with dementia, particularly in cases of chronic periodontitis [44,45]. Furthermore, Peptoniphilus, an opportunistic pathogen, is frequently identified as the principal causative agent of inflammation resulting from diverse infections, notably chronic wounds like diabetic foot ulcers and chronic compression [46]. This pathogen is also linked to the energy metabolism, leading to the production of the short-chain fatty acid butyrate [47,48]. Also, colonic microbes have the capability to synthesize B vitamins, supply nutrients to both the host and microbiota, and influence immune cell activity, as well as impact the nervous system through the production of neurotransmitters [16]. Thus, based on the flora screened, MDNs may affect cognitive function through a variety of pathways such as the modulation of neuroinflammation and immunity, the maintenance of bile acid homeostasis, the provision of OCM nutrients, energy metabolism, and other effects through the gut microbiota. The full extent of the functions of these flora remains unknown, given the evolving understanding of the gut microbiome in medical contexts. Consequently, the intricate pathways through which gut flora influence brain health have yet to be fully elucidated, necessitating the further exploration of the specific regulatory mechanisms involved.

Hence, in order to seek the potential pathways through which dietary MDNs may impact cognitive function via the gut microbiota, we utilized gut microbiomics for gene function annotation. In our study, it was observed that metabolic pathways associated with OCM, including Folate biosynthesis, Sulfur metabolism, Taurine and hypotaurine metabolism, and Glutathione metabolism, among others, were found to be significantly enriched in the D group. Conversely, the Cysteine and methionine metabolism pathway exhibited significantly lower enrichment in this group. Interestingly, these findings indicate that a deficiency in MDNs may lead to inadequate substrates for crucial reactions in the OCM pathway, subsequently increasing the body’s requirement for MDNs and prompting compensatory alterations in OCM-related pathways [49]. Additionally, the heightened oxidative stress and buildup of harmful substances associated with neurodegenerative conditions could amplify the body’s response to stress [50,51]. These indicate that the disruption of the complex regulatory network responsible for maintaining the OCM due to a deficiency in any individual MDN contributes to the accelerated progression of CI [16,52].

**Figure 6:**
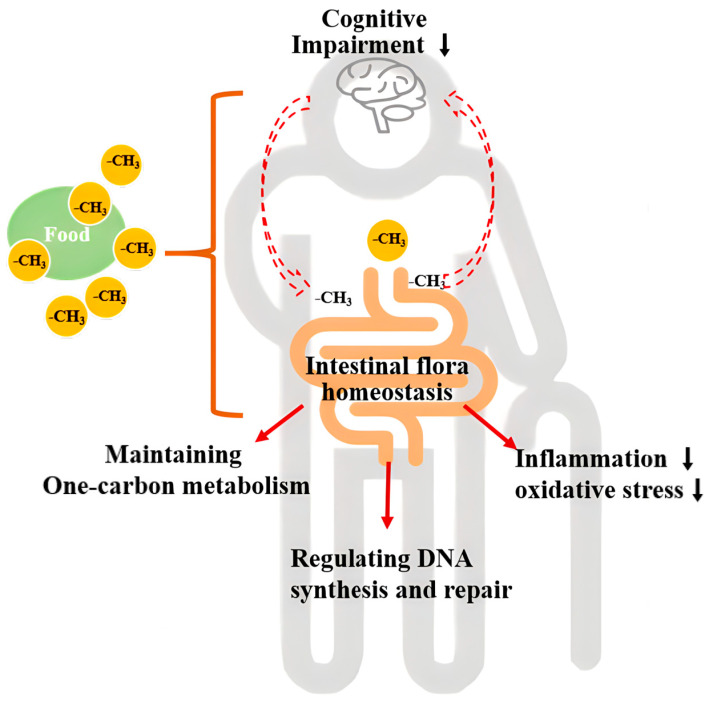
The potential mechanisms of dietary MDNs for cognitive function improvement through gut microbiota.

Additionally, some studies have shown that there are interactions between MDNs (folate and VB_12_) and the gut microbiota. Specific flora within the gut have been found to contribute significantly to the daily intake of folic acid, as well as various B vitamins including biotin, cobalamin, niacin, pantothenate, pyridoxine, riboflavin, and thiamine [53]. Conversely, diets lacking in folate and VB_12_ over an extended period can result in the reduced production of B vitamins by gut bacteria [25]. Based on the previous finds [25,53], the research indicates that a deficiency in MDNs may lead to a reduction in B vitamin-producing bacteria, which might impaire OCM in brain tissue and subsequent impacts on the nervous system. Notably, the significantly higher gene enrichment of the Lipopolysaccharide biosynthesis pathway was observed in the D group compared to the DF group. Porphyromonas was more abundant in HQ individuals compared to LQ individuals. It has been reported that folic acid supplementation reduces gut inflammation and mitigates oxidative stress and neurotoxicity, ultimately protecting neurons from damage [54]. Thus, it is suggested that high intake levels of MDNs may potentially help to slow down the progression of CI through reducing inflammatory flora, thereby attenuating chronic neuroinflammation, which was accordant with the previous report [54]. Furthermore, our study revealed a decrease in the gene enrichment of six pathways associated with DNA synthesis and repair, specifically Mismatch repair, Homologous recombination, DNA replication, Pyrimidine metabolism, Base excision repair, and Nucleotide excision repair, in the D group as compared to the DF group. Additionally, it is important to note that DNA synthesis and repair processes are intricately linked to OCM [55]. In a state of folate deficiency, the aberrant activation of serine hydroxymethyltransferase 1 (SHMT1) disrupts thymidylate biosynthesis, potentially resulting in cognitive dysfunction [56]. Taken together, the study may provide some clue that the modulation of intestinal flora homeostasis, the maintenance of OCM, the reduction in inflammation and oxidative stress, and the regulation of DNA synthesis and repair by MDNs may have a positive effect on cognitive function (see Figure 6 for illustration). Nonetheless, the intricate interplay among these pathways remains incompletely understood, necessitating additional mechanistic investigations.

Furthermore, in order to illustrate the significance of early intervention, an analysis was conducted on the distribution of the MNQI among D, MCI, and normal cognition (NC) populations. The results from the Supplementary Material revealed that over half of the individuals assessed were at risk of developing MCI, indicating a substantial portion of the population at heightened risk for dementia [57]. Moreover, there was no statistically significant difference between the MCI and NC groups in the MNQI, which was reflected as a mildly lower MNQI in the MCI group than the NC group. In contrast, the MNQI of the D group was significantly lower than both the MCI and NC groups. This, in conjunction with the reversible nature of MCI, underscores the significance of early intervention with MDNs during the MCI stage to mitigate the risk of progression to dementia [2]. In addition, our study revealed that the consumption of MDNs among the elderly fell significantly below the recommended levels (see Supplementary Tables S2 and S3). This deficiency of MDNs may be particularly pronounced in the neurological system as digestive and absorptive capabilities weaken with age. Consequently, it is advised that elderly individuals consider taking appropriate preventive measures, such as supplementation with MDNs, to potentially mitigate the decline in cognitive function.

We applied the MNQI for the first time to assess the relationship between the MDNs’ intake and cognitive function in older adults and explored the potential mechanisms of the gut microbiome, and screened for specific genera of bacteria, which has rarely been reported in prior studies. The present study initiatively utilized the MNQI for the assessment of the association between the intake of MDNs and cognitive function in elderly individuals. Additionally, the investigation delved into the potential mechanisms involving the gut microbiome and conducted a screening for specific bacterial genera, a topic that has received limited attention in prior research. Nevertheless, this study is constrained by certain limitations. Specifically, the sample size and study design are inadequate, leading to limited statistical validity, particularly in the realms of bacterial screening and functional prediction. Moreover, for community-based studies, this study is unable to obtain conclusive causation and segmented clinical diagnoses or treatment recommendations, as in clinical trial studies. Consequently, the present findings primarily serve as a foundation for future research. To more precisely elucidate the dynamic interplay between the nutrient intake, gut microbiota, and cognitive function, it is advisable to conduct future longitudinal studies with larger sample sizes. Furthermore, the inability to obtain the serum exposure levels of MDNs in the current study due to methodological constraints necessitates careful consideration in future research endeavors. Additionally, it is recommended that upcoming studies integrate multi-omics data to elucidate the effect of the gut microbiota on cognitive function, including its role in processes such as one-carbon metabolism, nutrient metabolism, immune modulation, and DNA synthesis. Ultimately, the gut flora identified through the screening conducted in this study offers preliminary insights for directing gut flora interventions aimed at enhancing cognitive function. It is advisable that future research endeavors encompass gut flora-focused animal or population intervention trials to substantiate their efficacy.

## 5. Conclusions

In conclusion, our research offers initial findings on the possible relationship between MDNs’ consumption and CI in the elderly, highlighting the significant influence of the gut microbiota composition and diversity in this correlation. The study underscores the importance of the early adoption of MDNs to preserve cognitive function in older individuals, and identifies specific gut flora strains that may provide positive help to the cognitive performance. These findings will help us obtain a deep understanding of the intricate relationship between the gut microbiota balance and neurological well-being in the aging population.

## Figures and Tables

**Table 1:** Demographic characteristics between dementia (D) and dementia-free (DF) groups.

Characteristics	D (*n* = 45)	DF (*n* = 245)	*p*-Value
Gender, male (%)	13 (28.9)	74 (30.2)	0.860
Age (years) x ± s	65.44 ± 2.72	65.67 ± 2.66	0.604
Highest educational level (*n*) %			
Primary school or below	4 (8.9)	18 (7.3)	0.958
Junior high or above	41 (91.1)	227 (92.7)	
Living alone or not (*n*) %			
Yes	6 (13.3)	19 (7.8)	0.245
No	39 (86.7)	226 (92.2)	
BMI (*n*) %			
Normal	16 (35.6)	119 (48.6)	0.108
Overweight or obese	29 (64.4)	126 (51.4)	
Chronic diseases history (*n*)%			
Detected	9 (20)	68 (27.8)	0.279
Not detected	36 (80)	177 (72.2)	

**Table 2:** Differences in MDNs intake between dementia (D) and dementia-free (DF) groups.

	D (*n* = 45)	DF (*n* = 245)	*p*-Value
MNQI	5.84 ± 3.13	6.86 ± 3.21	0.030 *
Protein	59.38 ± 25.40	71.64 ± 36.97	0.033 *
Folate	319.53 ± 194.05	375.93 ± 246.79	0.093
Choline	287.36 ± 123.06	327.51 ± 141.19	0.016 *
Riboflavin	0.88 ± 0.47	1.01 ± 0.52	0.017 *
VB_6_	2.02 ± 0.86	2.17 ± 1.08	0.342
VB_12_	1.80 ± 1.21	2.05 ± 2.28	0.595
Betaine	73.86 ± 63.31	75.44 ± 68.96	0.922
Zinc	7.31 ± 3.99	8.71 ± 4.94	0.028 *

* *p* < 0.05, according to the Mann–Whitney U test.

**Table 3:** Correlations between the relative abundance of screened genera and MoCA, MNQI, and MDNs’ intake.

CLR-Transformed Abundance		vs. Quality Score or Nutrient Intake									
		MoCA	MNQI	Protein	Folate	Choline	Riboflavin	VB_6_	VB_12_	Betaine	Zinc
Victivallis	r	0.127	0.048	0.060	0.074	0.116	−0.023	0.070	−0.047	0.130	0.018
	*p* value	0.030 *	0.410	0.312	0.208	0.048 *	0.696	0.236	0.422	0.028 *	0.766
Turicibacter	r	0.100	0.086	0.084	0.132	0.068	0.019	0.049	−0.047	0.237	−0.001
	*p* value	0.088 *	0.144	0.152	0.024 *	0.248	0.744	0.404	0.420	<0.001 *	0.982
Porphyromonas	r	−0.172	−0.092	−0.061	0.025	−0.019	−0.123	−0.079	−0.114	0.011	−0.071
	*p* value	0.004 *	0.118	0.304	0.668	0.744	0.036 *	0.182	0.052 *	0.848	0.228
Peptoniphilus	r	−0.103	−0.094	−0.122	−0.066	−0.116	−0.110	−0.074	−0.173	−0.073	−0.060
	*p* value	0.082 *	0.108	0.038 *	0.266	0.048 *	0.060 *	0.212	0.004 *	0.214	0.308
Howardella	r	−0.103	−0.094	−0.017	−0.025	−0.085	−0.043	−0.101	−0.108	−0.066	−0.027
	*p* value	0.080 *	0.110	0.778	0.666	0.150	0.464	0.086 *	0.068 *	0.264	0.646
Phascolarctobacterium	r	0.102	0.075	0.060	0.060	0.019	0.037	−0.015	0.038	0.097	0.013
	*p* value	0.082 *	0.200	0.306	0.308	0.744	0.526	0.794	0.524	0.098 *	0.820
Snodgrassella	r	0.135	0.076	0.120	0.055	0.077	0.106	0.031	0.049	0.075	0.058
	*p* value	0.022 *	0.200	0.040 *	0.348	0.190	0.072 *	0.596	0.404	0.204	0.324
Terrimonas	r	0.126	−0.001	0.094	0.106	−0.035	−0.003	0.031	−0.102	0.036	0.046
	*p* value	0.032 *	0.986	0.110	0.070 *	0.550	0.954	0.600	0.084 *	0.544	0.432
Planomicrobium	r	0.109	0.014	0.105	−0.019	0.104	−0.003	0.051	0.039	0.080	0.091
	*p* value	0.064 *	0.810	0.076 *	0.746	0.078 *	0.960	0.390	0.512	0.174	0.122
Centipeda	r	0.098	0.035	0.124	0.039	0.070	0.088	0.090	−0.024	0.082	0.097
	*p* value	0.098 *	0.548	0.034 *	0.512	0.234	0.132	0.126	0.680	0.166	0.100 *

* *p* < 0.1, according to the Spearman rank correlation analysis test.

## Data Availability

The original contributions presented in the study are included in the article/supplementary material. Due to specific legal restrictions, the data are not publicly available, and further inquiries can be directed to the corresponding author.

## Supplementary Materials

The following supporting information can be downloaded at: https://www.mdpi.com/article/10.3390/nu16132061/s1, Figure S1: Differences in MNQI among the D, MCI, and NC groups; Table S1: Demographic characteristics among the D, MCI and NC groups; Table S2: Evaluation of MDNs intake aged 60–64 years; Table S3: Evaluation of MDNs intake aged 65–70 years.

## Abbreviations

OCM	one-carbon metabolism
D	dementia
DF	dementia-free
MCI	mild cognitive impairment
AD	Alzheimer’s disease
MNQI	methyl-donor nutritional quality index
MDNs	methyl donor nutrients
PAD	photo-assisted dietary intake assessment
HQ	high methyl-donor nutritional quality
LQ	low methyl-donor nutritional quality
MoCA	Montreal Cognitive Assessment
OTUs	operational taxonomic units
BMI	body mass index
KEGG	Kyoto Encyclopedia of Genes and Genomes
rRNA	ribosomal ribonucleic acid
VB	vitamin B
PCR	polymerase chain reaction
PLS-DA	partial least squares discrimination analysis
Aβ	amyloid-β
SHMT1	hydroxymethyltransferase 1
