# Supplementary material for: Association of Methyl Donor Nutrients’ Dietary Intake and Cognitive Impairment in the Elderly Based on the Intestinal Microbiome
Source: Nutrients. 2024 Jun 28;16(13):2061. doi: 10.3390/nu16132061 (PMC11243070; doi:10.3390/nu16132061)
Supplement: Supplementary file 1 [file nutrients-16-02061-s001.zip › nutrients-3058216-supplementary.pdf]

Supplementary materials

Supplementary Figure S1. Differences in MNQI among the D, MCI, and NC groups.

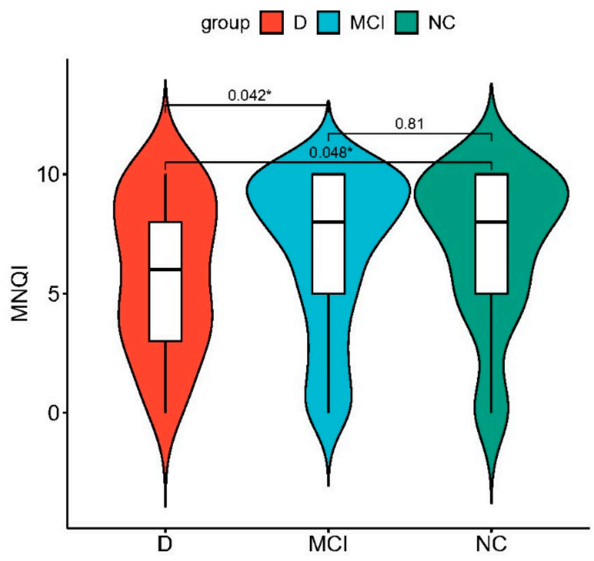

* P < 0.05, according to the Kruskal-Wallis test. Abbreviations: D, dementia; MCI, mild cognitive impairment; NC, Normal cognition; MNQI: methyl-donor nutritional quality index.

Supplementary Table S1. Demographic characteristics among the D, MCI and NC groups.

Characteristics	D (n=45)	MCI (n=190)	NC (n=55)	P-value
Gender， male (%)	13(28.9)	55(28.9)	19(34.5)	0.716
Age (years) x±s	65.44±2.72	65.59±2.71	65.93±2.50	0.629
Highest educational level (n) %				
Primary school or below	4(8.9)	14(7.4)	4(7.3)	0.940
Junior high or above	41(91.1)	176(92.6)	51(92.7)	
Living alone or not (n) %				
Yes	6(13.3)	12(6.3)	7(12.7)	0.170
No	39(86.7)	178(93.7)	48(87.3)	
BMI (n)%				
Normal	16(35.6)	93(48.9)	26(47.3)	0.268
Overweight or obese	29(64.4)	97(51.1)	29(52.7)	
Chronic diseases history (n)%				
Detected	9(20)	51(26.8)	17(30.9)	0.464
Not detected	36(80)	139(73.2)	38(69.1)	

Differences in gender, highest educational level, living alone or not, BMI and Chronic diseases history between groups were examined by Pearson's chi-square test. Differences in age between groups were examined by the analysis of variance (ANOVA) test.

Supplementary Table S2. Evaluation of MDNs intake aged 60-64 years[a].

MDNs	EAR		RNI/AI		UL
	Male	Female	Male	Female	
Protein(g)	60	50	65	55	—
Folate(ug DFE)[b]	320	320	400	400	1000
Folate(mg)	—	—	450	380	3000
Riboflavin(mg)	1.2	1	1.4	1.2	—
VB6(mg)	1.3	1.3	1.6	1.6	55
VB12(ug)	2	2	2.4	2.4	—
Zinc(mg)	10.1	6.9	12	8.5	40

[a] The population included in this study was in the age group of 60-70 years old, and the age group of 60-65 years old was referred to the data of the age group of 50-65 years old in the “Dietary Nutrient Reference Intake for Chinese Residents (2023)”;

[b] Dietary folate equivalent (DFE,μg) = natural food source folate (μg) + 1.7*synthesized folic acid[c] (DFE,μg);

[c] Refers to synthesized folic acid only, not including natural food source folate.

Supplementary Table S3. Evaluation of MDNs intake aged 65-70 years[a].

MDNS	EAR		RNI/AI		UL
	Male	Female	Male	Female	
Protein(g)	60	50	72	62	—
Folate(ug DFE)[b]	320	320	400	400	1000
Folate(mg)	—	—	450	380	3000
Riboflavin(mg)	1.2	1	1.4	1.2	—
VB6(mg)	1.3	1.3	1.6	1.6	55
VB12(ug)	2	2	2.4	2.4	—
Zinc(mg)	10.1	6.9	12	8.5	40

[a] The population included in this study was in the age group of 60-70 years old, and the age group of 65-70 years old was referred to the data of the age group of 65-75 years old in the “Dietary Nutrient Reference Intake for Chinese Residents (2023)”;

[b] Dietary folate equivalent (DFE,μg) = natural food source folate (μg) + 1.7*synthesized folic acid[c] (DFE,μg);

[c] Refers to synthesized folic acid only, not including natural food source folate.

Classification criteria:

A MoCA score of 18 or lower was categorized as the dementia (D) group and the rest were dementia-free (DF) group. In the DF group, a score of 25 or lower was graded as MCI group and the rest as normal cognition (NC) group[1].

Reference

[1] Tian, J.; Xie, H.; Wang, L.; Wang, Y.; Wang, H.; Shi, J.; Qin, B.; Fan, D.; Ni, J.; Sun, Y.; The Alzheimer's Disease Committee (ADC) Guideline Group of China National Health Care Association for the Elderly. Chinese guideline for the diagnosis and treatment of Alzheimer's disease dementia(2020). Chin. J. Geriatr. **2021**, *40*, 269-283, doi:10.3760/cma.j.issn.0254-9026.2021.03.001.
